# Viewing Trends and Users’ Perceptions of the Effect of Sleep-Aiding Music on YouTube: Quantification and Thematic Content Analysis

**DOI:** 10.2196/15697

**Published:** 2020-08-24

**Authors:** Ransome Eke, Tong Li, Kiersten Bond, Arlene Ho, Lisa Graves

**Affiliations:** 1 Department of Health Science University of Alabama Tuscaloosa, AL United States; 2 Western Michigan University School of Medicine Kalamazoo, MI United States

**Keywords:** insomnia, sleep deprivation, YouTube, utilization, pattern, perception, content analysis

## Abstract

**Background:**

Sleep plays an essential role in the psychological and physiological functioning of humans. A report from the Centers for Disease Control and Prevention (CDC) found that sleep duration was significantly reduced among US adults in 2012 compared to 1985. Studies have described a significant association between listening to soothing music and an improvement in sleep quality and sleep duration. YouTube is a platform where users can access sleep-aiding music videos. No literature exists pertaining to the use of sleep-aiding music on YouTube.

**Objective:**

This study aimed to examine the patterns of viewing sleep-aiding music videos on YouTube. We also performed a content analysis of the comments left on sleep-aiding music video posts, to describe the perception of users regarding the effects of these music videos on their sleep quality.

**Methods:**

We searched for sleep-aiding music videos published on YouTube between January 1, 2012, and December 31, 2017. We sorted videos by view number (highest to lowest) and used a targeted sampling approach to select eligible videos for qualitative content analysis. To perform the content analysis, we imported comments into a mixed-method analytical software. We summarized variables including total views, likes, dislikes, play duration, and age of published music videos. All descriptive statistics were completed with SAS statistical software.

**Results:**

We found a total of 238 sleep-aiding music videos on YouTube that met the inclusion criteria. The total view count was 1,467,747,018 and the total playtime was 84,252 minutes. The median play length was 186 minutes (IQR 122 to 480 minutes) and the like to dislike ratio was approximately 9 to 1. In total, 135 (56.7%) videos had over 1 million views, and 124 (52.1%) of the published sleep-aiding music videos had stayed active for 1 to 2 years. Overall, 4023 comments were extracted from 20 selected sleep-aiding music videos. Five overarching themes emerged in the reviewed comments, including viewers experiencing a sleep problem, perspective on the positive impact of the sleep-aiding music videos, no effect of the sleep-aiding music videos, time to initiation of sleep or sleep duration, and location of viewers. The overall κ statistic for the codes was 0.87 (range 0.85-0.96).

**Conclusions:**

This is the first study to examine the patterns of viewing sleep-aiding music videos on YouTube. We observed a substantial increase in the number of people using sleep-aiding music videos, with a wide variation in viewer location. This study supports the hypothesis that listening to soothing music has a positive impact on sleep habits.

## Introduction

Sleep plays an essential role in the psychological and physiological functioning of humans. Adults need 7 or more hours of sleep per night to be physically and mentally healthy [1]. The Centers for Disease and Control and Prevention (CDC) reports that sleep duration in adults substantially decreased from 1985 to 2012 [2]; in total, 35% of adults in the United States have a short sleep duration, which is defined as less than 7 hours per night [1]. Low sleep duration, or sleep deprivation, is associated with cognitive issues (including decreased task performance) and health issues such as diabetes, depression, obesity, cardiovascular disease, decreased cognitive performance, and decreased immune function [3-9].

Chronic sleep deprivation could be due to sleep disorders, such as insomnia. Insomnia includes difficulty falling asleep, trouble staying asleep, waking up too early, and poor quality of sleep [10], which directly affects an individual’s sleep duration. Insomniacs (persons diagnosed with insomnia) have higher rates of work absenteeism, decreased quality of life, and increased health care utilization when compared to good sleepers [11-13]. Insomnia can be treated with pharmacological, nonpharmacological, or both treatment methods, depending on the factors that trigger insomnia for that individual [14].

Nonpharmacological therapies are useful for increasing total sleep time or decreasing sleep onset latency and could help cut down on health care utilization costs and also offer fewer side effects when compared to pharmacological methods [14]. One nonpharmacological approach that appears to be effective in improving sleep quality is listening to music. Previous studies have found a significant association between an improvement in sleep quality and sleep duration and listening to soothing music [15,16]. Further, studies show that listening to soothing music for 45 minutes before bedtime may facilitate relaxation of the body and decrease serum cortisol by reducing stress, leading to improved quality of sleep in adults experiencing insomnia [17,18].

YouTube is a popular video-sharing platform, and the third most visited social media site in the world. Users can upload their own videos to YouTube, including sleep-aiding music videos. This platform allows users to share comments such as life experiences and relevant health information [19-22]. Published commentary about YouTube indicates that general usage increased about tenfold in 2017 compared to 2012; furthermore, it is estimated that over 1 billion hours of videos are watched daily, with about 400 hours of video uploaded each minute [23]. This medium combines fundamental technical features with a community formation function, allowing content creators to upload their videos to YouTube, while the company enables the delivery of this content to millions of viewers.

To date, no data exists describing the viewership of sleep-aiding music videos posted on YouTube and their impact on the sleep quality of users. Therefore, the primary aim of this study is to examine user viewing patterns of sleep-aiding music videos posted on YouTube. Furthermore, this study aims to describe, through content analysis, the perceptions of users regarding the effect that listening to sleep-aiding music videos has on sleep quality.

## Methods

### Data Source

We obtained data for this study from videos and comments posted on YouTube from January 1, 2012, to December 31, 2017. For ethical research purposes, this study is considered a nonhuman subject and therefore was exempt from institutional review board review.

### Search Approach

We searched YouTube video titles and descriptions for sleep-aiding music videos. The following keywords and phrases were used to search the related music videos: sleep, sleeping, music, soothing, and relaxing. These keywords were searched using Boolean logic “AND” and “OR” connectors. First, we searched for the keywords in the YouTube video title. Second, we searched with “the exact phrase.” Lastly, we searched with “all of the words” without a specific order.

The most common phrases used to reflect sleep-aiding music videos in YouTube post titles and descriptions included “sleep music,” “sleeping music,” “soothing music,” “relaxing music,” and “music for insomnia.”

### Inclusion Criteria

We selected videos posted in the English language from January 1, 2012, to December 31, 2017, by a YouTuber (an unofficial term used to describe people who create content and upload a video to YouTube). We only included videos that allowed active commenting at that time.

### Exclusion Criteria

We excluded YouTube videos posted by marketing agents or videos that promoted commercial content. Other exclusion criteria are duplicate videos, live streaming videos, languages other than English, videos with inappropriate or offensive materials, and videos that had disabled comments or ratings on the post. Additionally, we excluded similar comments that appeared multiple times across different videos.

### Data Extraction

We extracted data for descriptive statistics with the free, publicly available YouTube Comment Scraper project created by Philip Klostermann. This web client, licensed under the Internet Software Consortium (ISC), is written in Node.js (an open-source development platform for executing JavaScript code server-side) and uses the YouTube comment application programming interface (API) module to gain access to the comments. Given a YouTube video URL, the client will request all comments for that video from the API. Details about the YouTube Comment Scrapper project coding for all personal and local use is provided elsewhere [24].

We added available videos that met the study’s inclusion criteria to a private playlist. We clicked through each video and downloaded the parameters in comma-separated values (CSV) format. Parameters include music video title, view count, date of publication, comment text, number of likes, and dislikes. We generated a macro and used a macro recorder function in Microsoft Excel (Microsoft Corp) to automate the repetitive data extraction process. The age of the music video was estimated using the publication date.

To obtain data for content analysis, we selected videos using targeted sampling. Targeted sampling is an iterative procedure that assesses video features at several points, allowing adjustment to obtain a final sample similar to that of the hypothesized target population [25]. We defined the target sample for this study as the most viewed sleep-aiding YouTube music videos posted between January 1, 2012, and December 31, 2017. First, we sorted music videos by view number (highest to lowest) using the sort option on YouTube and relevance to our study. Next, using a random number generator and targeted sampling approach, we selected the 20 most viewed sleep-aiding music videos from the available videos.

As observed in a previous study, the criterion of most views is appropriate because an evaluation of these videos is likely to have the most significant impact (eg, a large number of viewers and comments); thus, this is a reasonable approach to the first evaluation of the media source [26]. Finally, we imported the Excel file into NVivo for Windows (Version 12 Plus; QSR International), a mixed-methods data analysis software [27]. We used the Word Cloud feature in NVivo 12 Plus to observe the most commonly used terms in the music video titles as well as the comments to get a picture of emerging concepts and ideas (Multimedia Appendices 1 and 2).

### Data Analysis

Descriptive statistics were generated based on the total number of videos posted, year of publication, video length, length of time since a given video was posted, number of views, and number of likes or dislikes. We summarized categorical variables as frequency and percentages, while numerical variables were summarized as means and standard deviation. SAS (Version 9.4; SAS Institute) was used to conduct all descriptive analyses.

Based on the word cloud of frequently used words in comments, two trained coders (authors TL and RE) applied a method described in a previous publication by Burla and colleagues [28] to code the extracted text. The coders excluded replies to individual comments due to their sheer number and structural challenges to storing these comments. Next, the coders sorted comments by character length before reading through relevant comments. Coders focused only on comments that described a user’s personal experience when listening to the sleep-aiding music video. The coders used NVivo to organize study data by creating nodes, sorting and reordering nodes within the same level of hierarchy and ratings, and merging nodes into other existing or new nodes. This enabled us to rate the music videos independently and identify recurring themes and patterns. The coders reviewed the video comments independently and documented codes in an Excel spreadsheet. We used the Microsoft Excel macro function to merge and match the comments under each code. The codes were developed using an inductive reasoning approach and discussed among the research team. Further, we computed the total number of relevant comments for each code. In addition, the coders examined any disparities of judgment to reach a consensus resolution. Lastly, the codes were merged into broader categories, including the positive and negative connotations of the comments, such as perceived pleasing and displeasing experiences.

We compared coding generated between coders and performed intercoder reliability assessment. For reference purposes only, we compared our themes with the automated items generated by NVivo.

## Results

### Descriptive Statistics

**Figure 1:**
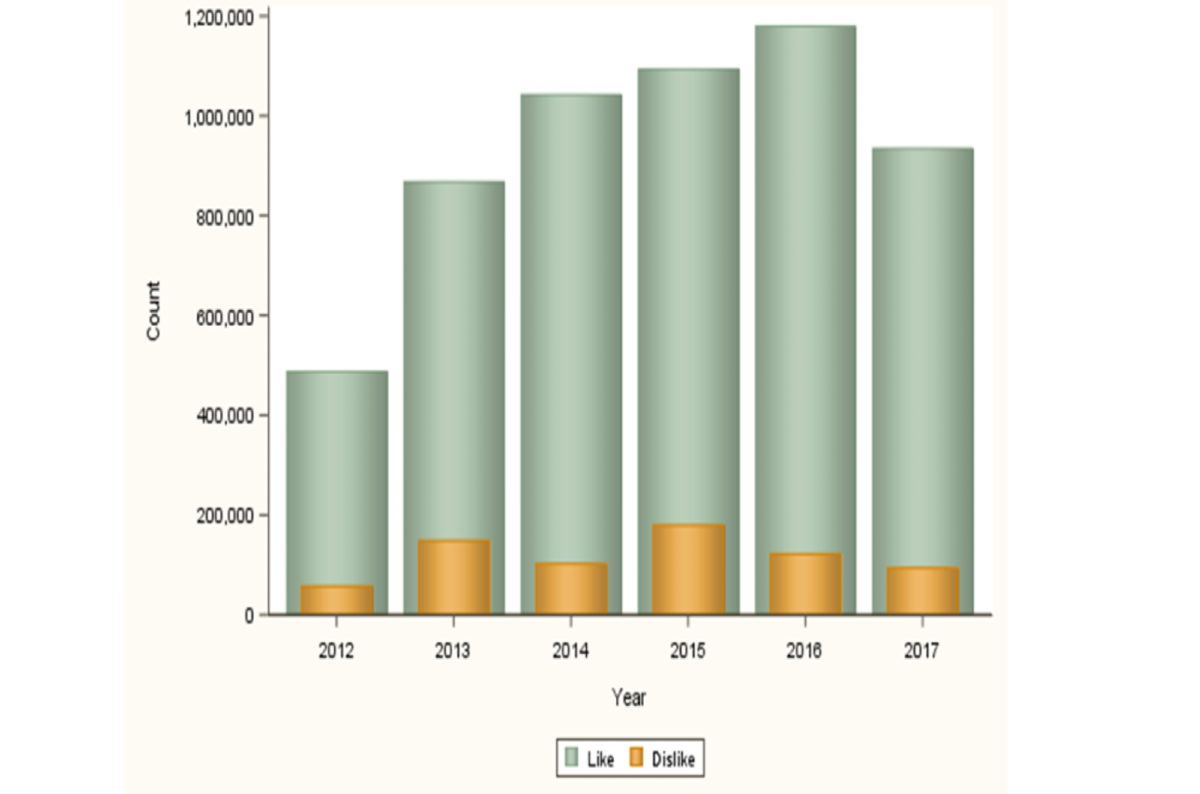
Comparison of the number of video likes versus dislikes by year of viewing.

After applying the filter option with the dates January 1, 2012, and December 31, 2017, we identified 238 YouTube sleep-aiding music videos that met the inclusion criteria for our study. Of the eligible music videos, there were a total of 1,467,747,018 view counts and a total playtime of 84,252 minutes. The median play length was 186 minutes (IQR 122 to 480 minutes), the like to dislike ratio was approximately 9 to 1 (Figure 1), 56.7% (135/238) had over 1 million views, and 52.1% (124/238) of the published sleep-aiding music videos had stayed active for 1 to 2 years (Table 1).

The proportion of total eligible music videos increased from 4.2% (10/238) in 2012 to 59.7% (142/238) in 2017. The average play length was 120 minutes and 25.6% (61/238) of the videos had been active for over 2 years.

**Table 1:** Summary of sleep-aiding YouTube music videos included in the study analysis (N=238).

Variable		Values, n (%)
**Length of music (minutes)**		
	<60	22 (9.2)
	60-120	32 (13.5)
	˃120	184 (77.3)
**Duration of post (years)**		
	<1	43 (18.1)
	1-2	124 (52.1)
	˃2	71 (29.8)
**Number of video views (million)**		
	<0.5	71 (29.8)
	0.5-1	32 (13.5)
	˃1	135 (56.7)
**Year posted**		
	2012	10 (3.4)
	2013	13 (4.4)
	2014	21 (7.1)
	2015	19 (6.5)
	2016	33 (11.2)
	2017	142 (48.3)

### Content Analysis

We considered only comments from 20 sleep-aiding music videos selected via the targeted sampling approach (Multimedia Appendix 3). The chosen videos had at least 100 comments relevant to our study. Five overarching themes emerged in the reviewed comments (Table 2): experiencing sleep problem, perspective on the positive impact of the sleep-aiding music videos, the perspective of no effect of the sleep-aiding music videos, opinion of time to initiation of sleep or sleep duration, and location of viewers. Table 3 describes the coding frequency for coded themes and interrater agreement rates. The overall κ statistic for the codes was 0.87 (range 0.85-0.96).

**Table 2:** Classification of themes from YouTube sleep-aiding music video comments.

Theme	Definition
Experiencing a sleeping problem	This theme is an indication of the viewers' perception of whether they are experiencing sleep deprivation or insomnia
Perspective on the positive impact of the sleep-aiding music videos	Content that serves as an indication that listening to the sleep-aiding music video on YouTube helped the viewer to sleep
The perspective of no impact of the sleep-aiding music videos	Content that serves as an indication that listening to a sleep-aiding music video on YouTube did not help the viewer to sleep or did not have any effect on sleep
The opinion of time to initiation of sleep or sleep duration	Content that serves as an indication of how long it took the viewer to sleep or how long the viewer slept after listening to the sleep-aiding music video (ie, viewer slept within minutes or had a longer sleep duration while listening to the music video)
Variation in the location of viewers	Content that serves as an indication of where the viewer is located while listening to the sleep-aiding music video

**Table 3:** Comments coding frequency, agreement rate, and percentage of nonassignment by coders^a^.

Code	Coding description	Coding frequency, n	Agreement rate (%)	Nonassignment caused by coder 1 (%)	Nonassignment caused by coder 2 (%)
001	Indication of whether the viewer expressed having a problem with sleeping (deprivation or insomnia)	670	69	57	43
002	Positive experience with viewing sleep-aiding music video on YouTube (listening to the music helped the viewer sleep)	2805	86	61	39
003	Negative experience with viewing sleep-aiding music video on YouTube (did not help the viewer sleep, did not have any effect on sleep)	437	74	48	52
004	Indication of how long it took the viewer to sleep or duration of sleep while listening to the sleep-aiding music video (ie, viewer slept within minutes or had a longer sleep duration)	786	93	50	50
005	An indication of whether the viewer mentioned his or her location in the comment	1236	100	65	35

^a^The data encompasses 20 selected music videos and 4023 comments. Overall code agreement rate: 84%. Intercoder reliability for all 5 codes: κ=0.87.

### Experiencing Sleeping Problem

Within this theme, viewers’ comments expressed whether they were experiencing any difficulty with sleeping, such as insomnia, or an inability to sleep or maintain an adequate duration of sleep. Over 16.6% (670/4024) of the comments reviewed contained messages expressing trouble sleeping. Additionally, some comments (362/4024, 9.0%) described the severity of the sleep problem. Some viewers' comments (604/4024, 15.0%) indicated the use of music videos for babies or children experiencing sleep difficulty. A sample of coded comments is presented here:

Thank you for sharing this! I suffer from insomnia and anxiety. My doctor prescribed meds to help me sleep. This has a calming effect and helps me sleep. Thank you so much!

Ref 8

I literally cannot fall asleep unless i listen to this. works every time

Ref 1

I am a sufferer of insomnia for more than two years and I thought the problem cannot be resolved. This sleep plan …. was suggested to me by a cognitive behavioral therapist. It totally changed the way I think about rest. I`m now sleeping comfortably again every night. My bed is now my friend again.

Ref 5

I have major depressive disorder and generalized anxiety disorder which has led to crippling insomnia-your videos are the only thing that help me sleep! …... Thank you for these videos! Love and peace to you!

Ref 14

I was diagnosed with GAD (General Anxiety Disorder) one of the problems I had with my anxiety was restless nights. This channel has helped me INCREDIBLY. Lack of sleep is killer, and it felt like I was going insane……back to my normal sleep cycle. I thank this channel so much. I’m truly happy again:)

Ref 20

Hi. I'm an insomniac mother with a newborn and almost 1 year old. We've all used this video. If they are crying and can't sleep I put this on.!

Ref 14

### Perspective on the Positive Impact of the Sleep-Aiding Music Videos

The effectiveness of listening to sleep-aiding music videos on YouTube was a dominant theme in our analysis. Most of the comments from viewers (2805/4023, 69.7%) suggested listening to the YouTube sleep-aiding music videos had a positive impact on their sleeping problem and sleep quality. Examples of comments in this theme include the following:

I discovered this music after being in the hospital for 4 days with viral meningitis. I got home extremely tired and couldn't fall asleep. After half an hour of no sleep, I tried searching up relaxing music….. I remember instantly falling asleep to this beautiful music. 9 months later I was struggling to get to sleep. I searched up relaxing music …... I've put it on ever since then and I can't sleep without it…. Good night from Canada

Ref 2

turned this on after 4 days of insomnia and working my tail off to wear myself out to no avail played less the five minutes of it and I feel completely drained and relaxed I think I may actually be able to sleep now…. Simply amazing how quickly it worked on me. Thank you

Ref 6

Omg you have no idea how good this works, every night I turn this on and fall asleep in minutes. FULLY RECOMMEND

Ref 20

### The Perspective of No Impact of the Sleep-Aiding Music Videos

On the contrary, not all viewers had a pleasant experience listening to YouTube sleep-aiding music videos. Some comments (437/4023, 10.9%) indicate that the music videos had no impact on their sleep problems or sleep quality.

I have a problem with sleeping, and this song doesn't work to make me sleep even when i am doing nothing but it can make me feel better,,, ….i have my own way to make me feel asleep although not always successful,…..because the people's problem is so heterogeneous and sometimes it isn't solved immediately.

Ref 1

Wide awake from CA. Guess soothing music doesn't work on insomnia, but worth a try, eh?

Ref 12

if you're reading this, then you are awake, which means this track is not working for you... on to the next one!!!

Ref 4

I still can't go to sleep with this music on

Ref 18

### Opinion About Time to Initiation of Sleep or Sleep Duration

Another important observation from the content analysis was the time it took viewers to sleep or how long they slept while listening to the sleep-aiding music videos. Information from analyzed comments (785/4023, 19.5%) indicated that time to initiation of sleep was between 5 and 30 minutes, and some viewers reported having 10 or more hours of sleep while listening to the music videos:

I fell asleep the first 8 freaking minutes

Ref 5

I actually fell asleep with this playing about 10 or so minutes into the video

Ref 18

I swear normally it takes me like one hour before i can get a shut eye.... so i came up with this and used it within 20 minutes i was in REM sleep...

Ref 5

As a person with insomnia, it's hard to fall asleep. Once I found this, I fell asleep within five minutes!!! Now that I have this, I went from only having 3-6 hours of sleep to 10-15 hours of sleep. The best part is I feel so refreshed in the morning!!!

Ref 1

### Variation of the Location of Viewers

Viewers frequently reported their place of viewing in some comments. YouTube is a widely accessible social media platform used worldwide; however, we were unable to get coordinate information to verify the location of users. Nevertheless, a word cloud of the comments showed that this was a significant theme, and 30.7% (1236/4023) of the comments expressed the area of viewers. The wide variation in the places mentioned in comments suggests that sleep-aiding music videos on YouTube are extensively used. For instance, the following comments describe users’ locations:

Goodnight from France

Ref 2

From US! Thank you for this amazing video! I've tried several different sounds to fall asleep as well as different methods……

Ref 8

I have anxiety, and I always go to sleep around 3 in the morning …..Normally I only get 4 or less hours of sleep …..Goodnight from Canada :)

Ref 9

Good morning from Italy! My family listens to it before to go to bed, and me, before arriving to work….Thank you!

Ref 17

Good nite from Philippines

Ref 19

Goodnight from Africa, thank you!

Ref 12

## Discussion

### Principal Findings

Our study shows that YouTube is a widely used social medium where viewers with sleep issues access sleep-aiding music videos to improve their sleep quality and habits. Dominant themes in the content analysis suggest that most of the users have a problem with sleeping such as insomnia; listening to the sleep-aiding music videos helps users to sleep within a short duration; and users have a more extended sleep period while listening to the sleep-aiding music videos. Further, the content analysis of comments revealed a wide variation in the location of viewers of sleep-aiding music videos published on YouTube.

This study provides evidence that suggests a growing number of people are accessing sleep-aiding music on YouTube. A body of literature has reported ever-increasing numbers of people suffering from insufficient sleep, as well as the effect of sleep deprivation on quality of life and productivity [29-31]. In the United States, a study found that there are between 50 and 70 million people with perceived chronic sleep or wakefulness issues. These numbers have increased over time, and over 35% of adults report having insufficient sleep [29,30]. Additionally, research on the effect of problems (both physical and psychosocial) related to sleeping difficulty and the treatment of sleep disorders has continued to gain attention in the past decade [32]. Findings from this study suggest that listening to sleep-aiding music videos on YouTube could be one of the approaches employed by people having some form of sleeping issues, both in the United States and around the world. A possible explanation for the increasing numbers of viewers using sleeping music videos could be that the rising cost of obtaining health care services and increasing difficulty in accessing health care is driving many individuals to less costly and more accessible alternatives to help with their sleep-related issues [33-35].

Recent studies have focused on health information acquisition from the internet and social media platforms, and aggregated data from these sources can provide useful public health information. For example, analyzing Twitter data was one of the approaches employed by the CDC to generate surveillance data during an influenza outbreak in the United States [32-34]. Results from this study suggest that over the 6-year study period, there was a substantial increase in the use of YouTube sleep-aiding music, and this could reflect a rising number of people who may have a sleeping disorder. Furthermore, our observations indicate that YouTube is a social support system where individuals with similar health or life experiences share information on important health topics impacting their health quality of life. Sleep quality is an essential component in the measures of quality of life.

There is a dearth of research examining the effect of frequent use of YouTube on sleep patterns. Therefore, we were unable to compare our findings with any similar research. Our study focused on the perceptions of viewers regarding the effectiveness of using YouTube sleep-aiding music videos to improve their sleep pattern. The results from our study show that most viewers perceive a positive effect of YouTube sleep videos on their sleep quality and duration. Nevertheless, it has been shown by several studies that the frequent use of social media adversely affects sleep quality and pattern among users [36-39]. A study conducted among Canadian youths showed the use of social media for at least 1 hour per day was associated with higher odds of short sleep duration in a dose-response manner [40]. A study by Levenson and colleagues [41] examined the independent association of social media use 30 minutes before bed and disturbed sleep. Their study found a significant linear trend in the odds ratios between the frequency of checking social media in the 30 minutes before bed and increased sleep disturbance. Even though our study result is not directly comparable with these reports, the comments obtained for our content analysis established that individuals with sleep disturbances use sleep-aiding music videos on YouTube to enhance their sleep habits and maintain adequate and prolonged sleep. This topic requires further exploration to understand how frequent use of YouTube could impact the sleep quality of users.

The capability of using social media to monitor public health issues by location is well documented [42-45]. For instance, Twitter provides location information for some tweets, aiding geographical biosurveillance of emerging health topics. During the recent Zika outbreak, communities worldwide discussed the disease and critical issues associated with it on Twitter. Data collected from tweets reflected the spread of interest in Zika from its original hotspot in South America to North America and then across the globe [43]. Unlike Twitter, YouTube does not provide the location coordinates of users. Still, users of YouTube could freely offer information about their location in comments. Data from our content analysis of comments include the varied geographic location of users of sleep-aiding music videos on YouTube. Observations from analyzed comments indicate that the users were located both in developed and developing countries. This finding also suggests that difficulty in sleeping is a global issue and supports the notion that social networking platforms can provide an understanding of the burden of health problems through spatial information from comments and the platforms’ geocoding systems.

This study provided valuable information on the pattern of use of sleep-aiding music videos on YouTube; the findings could indicate a significant health burden exists worldwide. However, this study had some limitations. First, the comments used for the content analysis were self-reported and we are unable to verify claims of sleeping problems such as insomnia. Second, our units of analysis for this study were the number of views and comments posted, and we were unable to assess individual-level data such as sociodemographic variables to compare differences between groups. In addition, YouTube users could have multiple accounts with privileges such as posting the same videos with different titles and commenting under different usernames. To minimize this issue in our analysis, we sorted and excluded videos with similar content, and any comments with the exact same wording that appeared multiple times across videos. Third, due to the volume of sleep-aiding music videos on YouTube and the large amount of comments posted, we used the targeted sampling approach to select samples from the most viewed videos. This approach could have eliminated some vital music videos, possibly resulting in our missing other essential themes. This limitation presents an opportunity for further research on this topic using a similar data source. Finally, the contents of YouTube videos are accessible to the public, and no formal approval is required to access the contents of the postings. Notwithstanding, users must register to upload videos or post comments on the site. Although the usernames in the comment section are publicly available, users are free to create multiple accounts and hold numerous pseudonyms. Therefore, we cannot guarantee that all the comments we extracted were genuine with no astroturfing.

### Conclusions

This study provides information regarding the use of YouTube sleep-aiding music videos to ameliorate sleep problems and improve sleep quality. We observed a substantial increase in the number of people using sleep-aiding music videos, and a wide variation in their location. This study also supports the positive impact of listening to soothing music on sleep habits. Finally, this study demonstrates that YouTube is an essential social medium for acquiring and analyzing crucial public health information.

## Appendix

Multimedia Appendix 1 Word cloud of the most frequent terms used in the music video titles.

Multimedia Appendix 2 Word cloud of the most frequent words used in comments. The larger words represent more frequently used terms, while the smaller words were less frequently used.

Multimedia Appendix 3 Description of videos randomly selected for thematic content analysis.

## Abbreviations

API: application programming interface
CDC: Centers for Disease Control and Prevention
