# Supplementary figures and images for: Viewing Trends and Users’ Perceptions of the Effect of Sleep-Aiding Music on YouTube: Quantification and Thematic Content Analysis
Source: J Med Internet Res. 2020 Aug 24;22(8):e15697. doi: 10.2196/15697 (PMC7477671; doi:10.2196/15697)

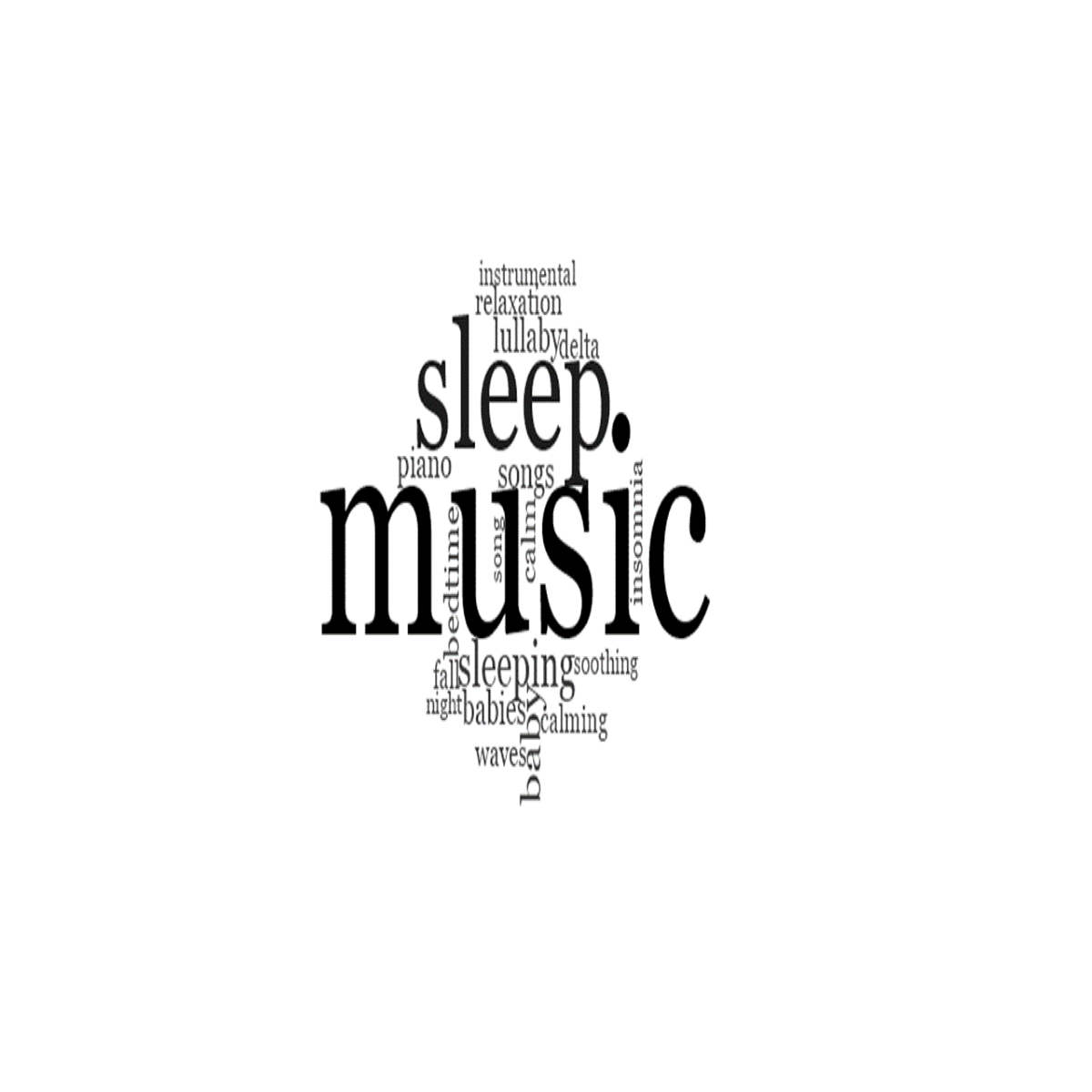

Supplement: Multimedia Appendix 1 [file jmir_v22i8e15697_app1.png]

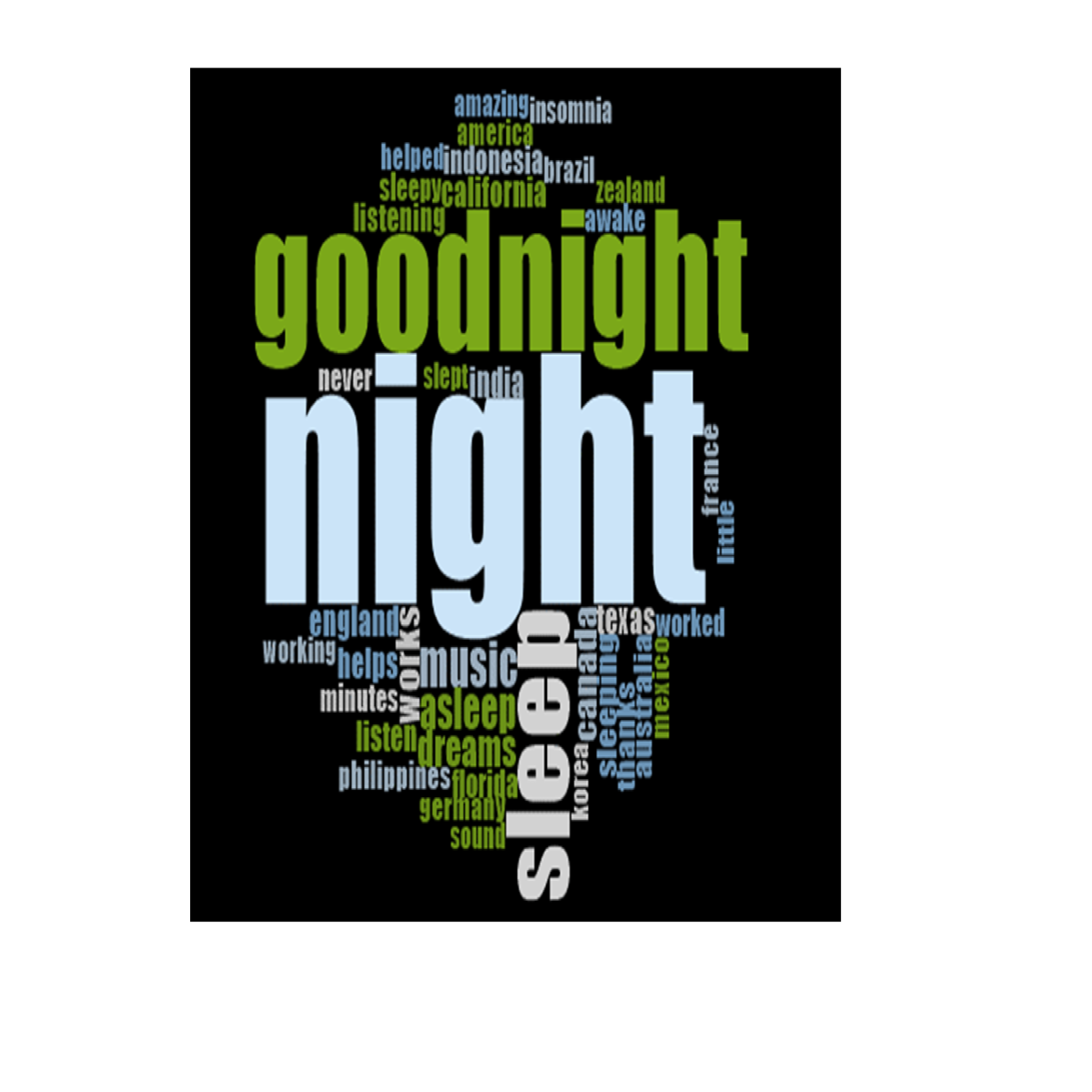

Supplement: Multimedia Appendix 2 [file jmir_v22i8e15697_app2.png]
