# Supplementary material for: Viewing Trends and Users’ Perceptions of the Effect of Sleep-Aiding Music on YouTube: Quantification and Thematic Content Analysis
Source: J Med Internet Res. 2020 Aug 24;22(8):e15697. doi: 10.2196/15697 (PMC7477671; doi:10.2196/15697)
Supplement: Multimedia Appendix 3 [file jmir_v22i8e15697_app3.docx]

**Supplementary Table 1: Description of videos randomly selected for thematic content analysis**

| Video Number | Title | Comments | Study relevant comments | Views | Like | Dislike | Publish Date | Duration |
| --- | --- | --- | --- | --- | --- | --- | --- | --- |
| 1 | Sleep Music Delta Waves: Relaxing Music to Help you Sleep, Deep Sleep, Inner Peace | 76747 | 270 | 95,394,468 | 339,470 | 37,693 | 11/16/2012 | 44.967 |
| 2 | Relaxing Sleep Music: Deep Sleeping Music, Relaxing Music, Stress Relief, Meditation Music ★68 | 27431 | 220 | 62,831,740 | 374,033 | 28,332 | 7/3/2016 | 180.350 |
| 3 | 8 Hour Deep Sleep Music: Delta Waves, Relaxing Music Sleep, Sleeping Music, Sleep Meditation, ☯159 | 17333 | 300 | 40,427,681 | 152,893 | 14,249 | 6/22/2014 | 479.483 |
| 4 | 30 Minute Deep Sleep Music: Calming Music, Relaxing Music, Soothing Music, Calming Music, ☯426B | 3699 | 210 | 17,482,973 | 70,237 | 7,417 | 9/23/2015 | 30.017 |
| 5 | 3 HOURS of Gentle Night Rain, Rain, Rain Sounds - Sleep, Insomnia, Meditation, Relaxing, Yoga, Study | 8578 | 302 | 13,814,250 | 86,033 | 5,834 | 11/25/2014 | 181.583 |
| 6 | Deep relaxing sleep music: Go for your deepest sleep yet. (3 hours) for Insomnia Help Calm Music | 2312 | 158 | 13,101,499 | 47,963 | 6,852 | 6/5/2015 | 179.783 |
| 7 | SLEEP MUSIC RELAXING MUSIC INSOMNIA HELP SLEEPING MUSIC MUSIC FOR DEEP SLEEP HELP | 4967 | 218 | 11,158,271 | 19,698 | 2,162 | 7/3/2009 | 578.000 |
| 8 | 8 HOUR Sleep Music Delta Waves: Relaxing Music, Beat Insomnia, Calming Music, Deep Sleep, ☯183A | 3442 | 208 | 10,814,251 | 38,234 | 3,827 | 7/9/2015 | 480.467 |
| 9 | 8 Hour Sleep Music For Insomnia: Deep Sleep Music, Sleeping Music, Help Insomnia ☯207 | 3664 | 340 | 10,750,593 | 48,912 | 5,511 | 8/6/2014 | 477.933 |
| 10 | Relaxing Sleep Music: Deep Sleeping Music, Fall Asleep Fast, Soft Piano Music, Ocean Waves ★104 | 2538 | 273 | 5,128,478 | 35,244 | 2,882 | 12/1/2016 | 182.733 |
| 11 | Relaxing Piano Music: Soft Sleep Music, Water Sounds, Meditation Music, Relaxing Music ★102 | 2291 | 154 | 4,533,778 | 28,736 | 2,090 | 11/8/2016 | 183.167 |
| 12 | 8 Hour Sleeping Music: Relaxing Music, Sleep Music, Deep Sleep, Relaxation Music, Insomnia, ☯992 | 1792 | 140 | 4,295,879 | 14,252 | 2,014 | 4/21/2015 | 480.467 |
| 13 | Relaxing Piano Music: Sleep Music, Meditation Music, Soothing Music, Calming Music ★77 | 1525 | 162 | 4,258,386 | 24,268 | 1,902 | 7/23/2016 | 184.067 |
| 14 | 8 Hour Sleep Music, Calm Music for Sleeping, Delta Waves, Relax, Insomnia, Relaxing Music, ☯2817 | 2353 | 184 | 3,495,775 | 14,365 | 1,716 | 6/28/2016 | 480.467 |
| 15 | Sleep Music: Sleeping Music and Relaxing Music for Sleeping,Relax,Lullabies to help you Sleep | 3034 | 198 | 3,192,486 | 7,484 | 1,001 | 10/5/2012 | 70.200 |
| 16 | Sleep Music:Sleeping Music for Deep Sleep Stress Relief Relaxing Sleep Sounds Bedtime Songs | 2122 | 180 | 3,061,535 | 8,398 | 1,167 | 10/10/2012 | 39.800 |
| 17 | 6 Hours of Relaxing Sleep Music: Deep Sleeping Music, Fall Asleep, Sweet Dreams ★68 | 2002 | 125 | 2,881,872 | 17,875 | 1,995 | 7/11/2016 | 360.633 |
| 18 | Fall Asleep Fast: Deep Sleep Music, Relaxing Music, Stress Relief, Sleeping Music ★130 | 3075 | 166 | 1,951,810 | 15,954 | 1,251 | 12/28/2017 | 186.533 |
| 19 | ULTIMATE DEEP SLEEP music- Healing INSOMNIA / 20 min of Sleep Relaxation | 681 | 103 | 1,918,247 | 7,343 | 870 | 5/8/2013 | 19.667 |
| 20 | SLEEPING MUSIC FALL ASLEEP FAST WITH RELAXING SOUNDS | 760 | 112 | 1,494,360 | 2,423 | 271 | 7/11/2009 | 591.000 |
